# Pancreatic Metastases of Esophageal Squamous Cell Carcinoma: A Case Report and Review of the Literature

**DOI:** 10.3390/diagnostics14192164

**Published:** 2024-09-28

**Authors:** Tivadar Bara, Alexandra Georgiana Scurtu, Tivadar Bara, Zsolt Zoltan Fulop, Renata Moriczi, Patricia Simu, Paul Borz, Simona Gurzu

**Affiliations:** 1 Department of 2nd Surgery, George Emil Palade University of Medicine, Science and Technology of Targu Mureş, 540139 Targu Mureș, Romania; btibi_ms@yahoo.com (T.B.J.); zsolt_fulop15@yahoo.com (Z.Z.F.); 2 Department of 2nd Surgery, Clinical County Hospital, 540136 Targu Mureş, Romania; barativadar@yahoo.com (T.B.); moriczi.renata@yahoo.com (R.M.); 3 Department of Radiology and Imaging, Clinical County Emergency Hospital, 540136 Targu Mureş, Romania; simupatricia@gmail.com; 4 Department of Pathology, George Emil Palade University of Medicine, Science and Technology of Targu Mureş, 540139 Targu Mureş, Romania; borz_paul96@yahoo.com; 5 Research Center of Oncopathology and Translational Medicine, George Emil Palade University of Medicine, Science and Technology of Targu Mureş, 540139 Targu Mureş, Romania; 6 Romanian Academy of Medical Sciences, 030167 Bucharest, Romania

**Keywords:** esophagus, squamous cell carcinoma, pancreatic metastasis

## Abstract

Esophageal carcinoma is an aggressive cancer with a poor therapeutic response and a significant risk of recurrence after radical resection. It usually metastasizes to the lung, bones, or liver. Unusual spread can be found in other organs, but only nine cases of pancreatic metastases have been reported in the Medline database. In the present paper, a literature review of nine cases with esophageal squamous cell carcinoma and pancreatic metastasis was carried out. In addition to these cases, we present our case, the tenth case in the literature. It involved a patient who underwent surgery for esophageal squamous cell carcinoma and developed metachronous pancreatic metastasis 67 months after esophagectomy. Histopathological examination confirmed a squamous cell carcinoma metastasis. Conclusions: Pancreatic metastasis of esophageal squamous cell carcinoma is extremely rare. Pancreatic metastasis may develop several years after the treatment of the primary lesion. The diagnosis of metastasis is difficult, requiring histopathological and immunohistochemical examination.

## 1. Introduction

Esophageal squamous cell carcinoma (ESCC) has a poor prognosis, with poor therapeutic results. It is known to have a high potential for metastasis and a significant risk of recurrence after radical resection [1,2,3,4,5,6]. Recurrence after radical esophagectomy is detected in 30–50% of patients with hematogenous or lymphatic recurrence [1]. Esophageal cancer, diagnosed at an advanced stage due to the inability to detect symptoms, is the ninth most common malignancy and the sixth most common cause of cancer mortality worldwide [6].

The most common metastases occur in the lymph nodes, lungs, liver, bones, adrenal glands and brain [1,2]. Recently, a few cases of unexpected metastasis with localization in uncommon organs, the so-called “unexpected localizations” (uncommon sites), have been reported [1]. Pancreatic metastasis (PM) in esophageal squamous cell carcinoma (ESCC) is an extremely rare entity. Since malignant tumors of the pancreas are usually primary neoplasms, pancreatic metastases (PMs) are very rare, accounting for approximately 2–5% of all pancreatic tumors [2,3,4,5,6,7,8]. Only 0.1–4.8% of PMs are caused by primary esophageal cancer [2,3,7,8]. Another study, conducted by Koizumi W, shows PMs caused by esophageal cancer with frequencies of 0.1% for esophageal adenocarcinoma and 2.9% for ESCC [8]. Similarly, autopsy studies have shown that 3–15% of pancreatic tumors are secondary lesions, and the rate of esophageal carcinoma PMs is 3.9–4.9% [7,8,9,10]. Unexpected PMs of ESCC are rare, being described in the literature only by sporadic case presentations [8].

## 2. Case Report

We present the case of a 75-year-old man with solitary metachronous pancreatic metastasis of ESCC. The patient underwent surgery in 2015 in our clinic for a poorly differentiated ESCC. The tumor was located in the lower one-third of the esophagus. We obtained an accurate medical history of the patient, and no pre-existing chronic conditions were found. The surgical intervention consisted of abdominal cervical esophagectomy with gastric tube reconstruction (the Akiyama procedure) [11]. No postoperative complications were recorded after ESCC treatment. The microscopy revealed ESCC extending beyond adventitia. Metastases in four of the seven regional lymph nodes (periesophageal, paracardiac) and three lymph nodes of the celiac axis were identified, along with angiolymphatic and perineural invasion.

After the esophagectomy, the patient refused the oncological therapy indicated by the oncology team. He also did not follow any treatment or diet.

**Figure 1:**
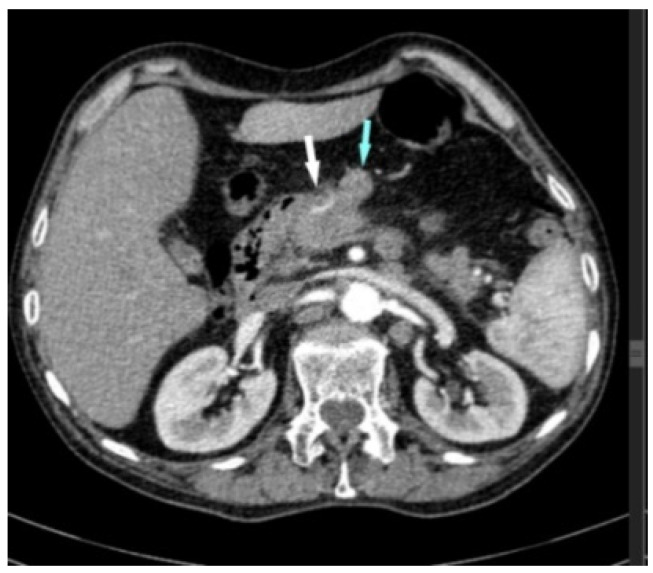
Contrast-enhanced CT: space-replacing process enclosing the gastroduodenal artery (white arrow) and a peripancreatic adenopathy (blue arrow).

**Figure 2:**
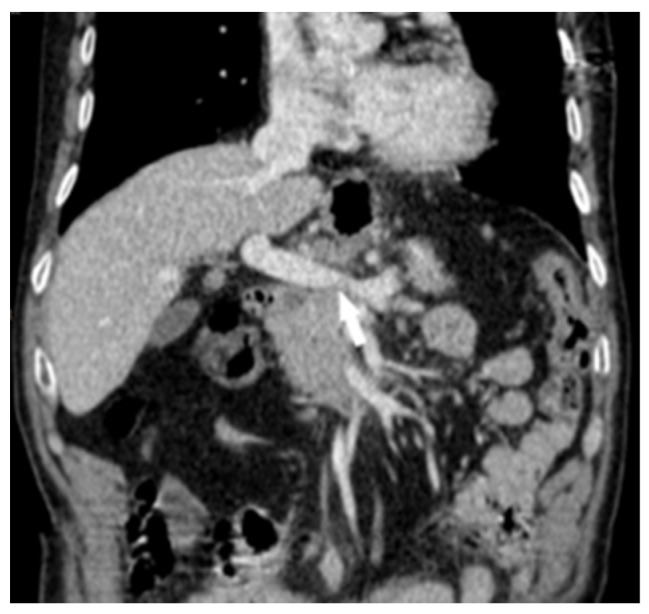
Contrast-enhanced CT: in the venous phase, the tumor process compresses the splenomesenteric confluence, causing the loss of the cleavage plane with the first portion of the portal vein (arrow).

**Figure 3:**
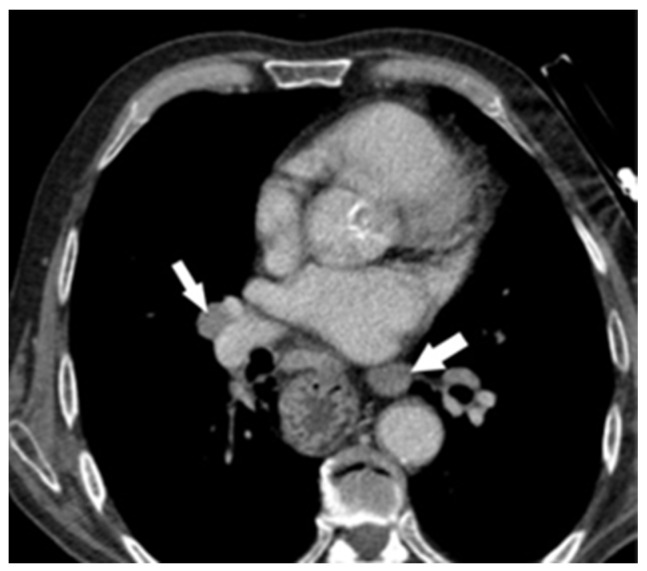
Contrast-enhanced CT—mediastinal and pulmonar hilar adenopathies (arrows).

In 2021, 67 months after esophagectomy, the patient was re-admitted to our clinic with a diagnosis of pancreatic tumor, complaining about weight loss of 10 kg and pain in the epigastrium and lumbar spine. He stated that the symptoms started about 3 months earlier. Laboratory examinations revealed mild normocytic anemia (hemoglobin: 7.9 g%; hematocrit: 27.2%). Tumor markers (CEA, CA 19-9) were within the normal limit. A contrast-enhanced computed tomography (CT) scan revealed a cephalic pancreatic tumor, with dimensions of an approx. 3.26/3.04 cm, infiltrative-type, imprecisely delimited, inhomogeneous hypovascular mass enclosing the gastroduodenal artery and the superior pancreaticoduodenal branches (Figure 1). The tumor compresses the splenomesenteric confluence, with apparent loss of the cleavage plane of the first portion of portal vein vein (Figure 2), and the scan reveals peripancreatic, paraaortic, inter-aortico-cave, lumbo-aortic and mediastinal and pulmonar hilar adenopathy (Figure 3).

**Figure 4:**
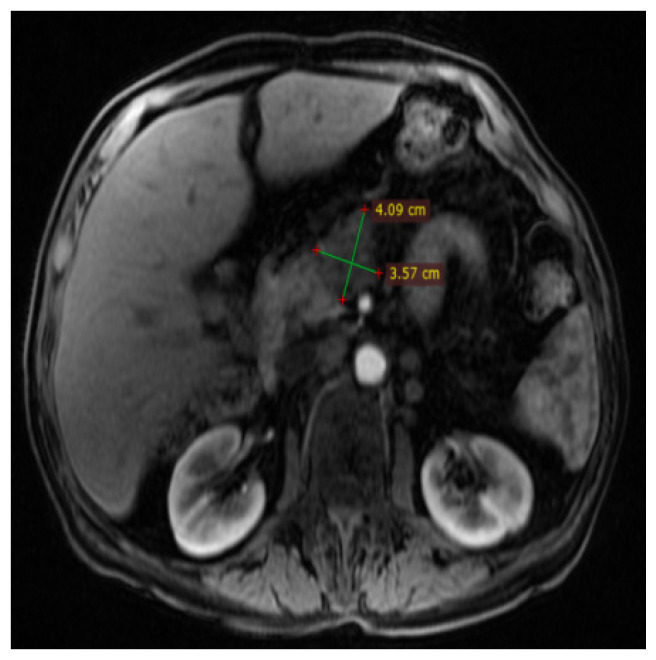
Contrast-enhanced MRI: in the arterial phase, a pancreatic tumor (dimensions of approx. 4.09/3.57 cm) with a lower contrast intake compared to the rest of the pancreas–hypovascular character was detected.

**Figure 5:**
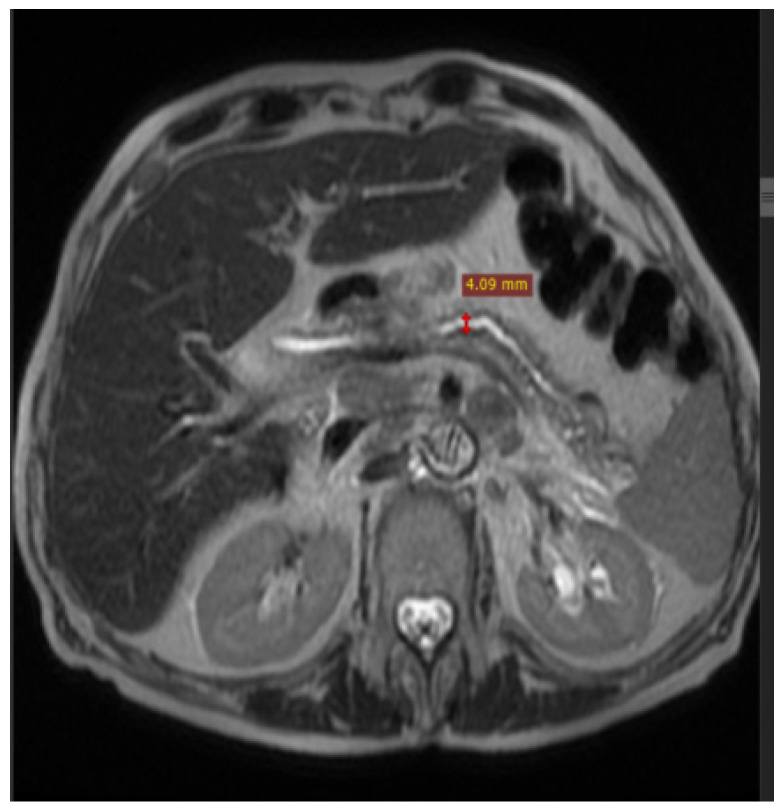
Contrast-enhanced MRI: the Wirsung duct was compressed by the cephalic pancreatic process, with its dilation increasing to 4.09 mm in the corporeocaudal portion.

**Figure 6:**
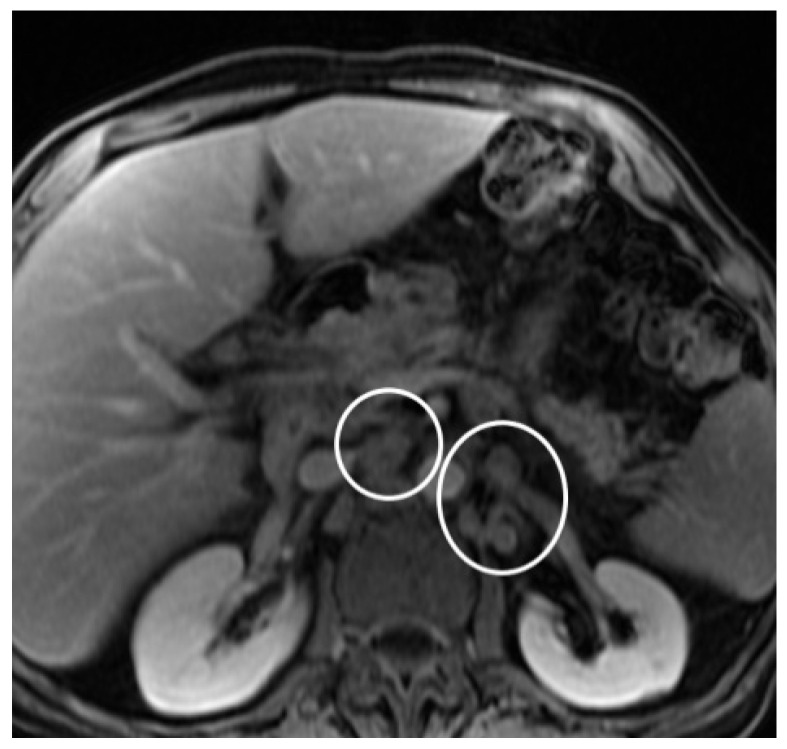
Contrast-enhanced MRI: the venous phase revealed multiple para-aortic and lumbo-aortic adenopathies (circles).

For additional information, contrast-enhanced Magnetic Resonance Imaging (MRI) was performed, describing the same inhomogeneous hypovascular tumor at the level of the pancreatic head, compressing and leaving no cleavage plane in relation to splenomesenteric confluent, with a filling defect in the adjacent portion of the superior mesenteric vein, suggesting thrombosis or invasion. The Wirsung duct was compressed by the cephalic pancreatic process, with distal duct dilatation occurring (Figure 4, Figure 5 and Figure 6).

Echoendoscopy and percutaneous puncture are not available in our hospital, only being available in another gastroenterology center in another city. Being in the middle of the COVID-19 pandemic, we were not allowed to send the patient to another medical center in another region to perform the endoscopic investigation; that is why the oncological committee decided that in order to confirm the histopathological diagnosis, the pancreatic biopsy should be performed through a surgical approach. We tried to perform pancreatic biopsy by a laparoscopic approach; however, for the first surgery (the abdominocervical esophagectomy) performed by an open approach after the introduction of a camera, we found extensive adhesions (between the omentum, intestinal loops, liver and abdominal wall). Considering the high risk of laparoscopy, we chose to perform pancreatic biopsy by an open approach. Intraoperatively, as the pancreatic mass was considered unresectable, we performed only pancreatic and lymph node biopsy.

The piece sent for histopathological examination was represented by a fragment of pancreatic parenchyma, autolyzed and infiltrated by islands and nests of tumor cells consisting of large, polygonal cells, similar to those in the spinous layer of the epidermis, with the foci of keratinization with nuclear pleomorphism. The islands of tumor cells showed desmoplastic stromas and areas of extensive necrosis and exceeded the resection margins. A tumor-infiltrated lymph node with perilymphonodular extension was also identified. The histopathological diagnosis was squamous cell carcinoma metastasis in the pancreas with extension in the peripancreatic lymph nodes and tumor emboli in the blood vessels. The postoperative evolution was favorable, with the patient being discharged on the sixth postoperative day with a recommendation for palliative oncological treatment.

After confirming the histopathological diagnosis of pancreatic metastasis of esophageal squamous cell carcinoma, the patient was referred to the territorial oncology service, but he refused the indicated oncological therapy and followed only symptomatic treatment, taking only painkillers, and unfortunately died after 7 months.

### Search Strategy

To perform a review of the previously reported PMs from ESCC, we analyzed all papers indexed in the Web of Science and Medline databases between 2011 and 2023, using the following keywords: esophageal squamous cell carcinoma and pancreatic metastasis, secondary tumors and pancreas.

Relevant articles were reviewed and sorted for final analysis. During the period under study, 10 articles with these keywords were found to be relevant [1,2,3,4,5,6,7,8,9,10], with 9 of them being case presentations [2,3,4,5,6,7,8,9,10]. Another 19 articles were included to acquire a full picture of the secondary tumors of the pancreas [12,13,14,15,16,17,18,19,20,21,22,23,24,25,26,27,28,29,30]. Data from these articles included baseline demographics, primary tumor histology, the interval between the diagnosis of esophageal carcinoma and diagnosis of PM, the localization of the metastasis in the pancreas, therapeutic management and survival. The first case of PM of ESCC was published by Esfehani et al. in 2011 [9]. Another eight case reports were subsequently published until 2023 (Table 1). Among these, six patients showed synchronous ESCC and PMs [3,4,5,6,7,10]. Two of the five patients also showed metastases in the vertebrae [4] and liver [7], respectively. In the other three cases, the metastases occurred more than six months after the diagnosis of ESCC and were considered to be metachronous PMs [2,8,9].

In only five of the nine cases, distal pancreatectomy was performed for solitary metachronous (*n* = 3) [2,8,9] or synchronous metastasis (*n* = 2) [3,10]. We included our case in this table as a tenth case about a patient who underwent surgery for esophageal squamous cell carcinoma and developed metachronous pancreatic metastasis 67 months after esophagectomy.

## 3. Discussions

Of all tumors that show a predisposition for pancreatic metastasis, renal cell carcinoma (RCC) ranks first. It accounts for 62.6–70% of cases, followed by melanoma (9.1%), colorectal cancer (8.8%), sarcoma (4.5%), breast cancer (4.3%) and lung cancer (3.1%) [2,3,4,14,31,32,33,34,35,36,37,38,39,40,41].

The first case of renal cancer with PM, known as hypernephroma, was published in 1952 by Jenssen et al. [42,43]. About 1470 cases of RCC with PMs were published between 1952 and 2023, most of them as case reports or case series [43]. Although uncommon, the number of cases with PMs is growing. They can be divided into two clinicopathological types: widespread systemic disease or an isolated lesion of the pancreas [22,23,30]. In daily practice, differentiation between a primary tumor of the pancreas and a PM is difficult to achieve [25,34,37]. However, in any patient with a history of extra-pancreatic malignancy, any pancreatic lesion should be suspected to be a PM [12,35,42]. PMs can develop synchronously along with the primary tumor or metachronously between six months and several years after the treatment of the primary lesion. The latest occurrence of pancreatic metastases after removal of the primary tumor was 11 years after ESCC and 36 years after RCC [8,13,23,24,26,42]. In our case, pancreatic metastasis of ESCC was discovered after 67 months since esophagectomy as a solitary metachronous tumor. Isolated PMs are rare and are often misdiagnosed as primary tumors [15].

PMs are asymptomatic in over 50% of cases, often being detected during oncological surveillance after surgery for a primary lesion or as an incidental finding during imaging studies performed for another disease [17,22,29,30]. Most patients with PMs do not present specific symptoms of metastasis, the clinical presentation of PM being similar to that of primary neoplasm, which makes diagnosis more difficult [29,42]. The most common symptoms are epigastric or back pain, weight loss, anorexia, nausea, vomiting, gastrointestinal bleeding and jaundice if the metastasis is located in the head of the pancreas [13,17,29,42]. Laboratory tests are nonspecific and cannot differentiate a primary pancreatic cancer from a PM [17].

Routine CT-scan imaging may be the most effective way to identify a PM [23,26]. However, tumor size, echogenicity, location and the number of lesions were not found to be significantly different between primary and metastatic tumors of the pancreas, independently of the imaging method (CT; MRI—Magnetic Resonance Imaging; Pet CT—Positron Emission Tomography; EUS—Endoscopic Ultrasonography) [7,13,19,23,36,43]. Primary adenocarcinoma may show hypo-amplification as opposed to the improved peripheral or homogeneous contrast that is seen in PM, but the differences are not significant [4,7,15,23,34]. On EUS, PMs tend to have more well-defined margins than primary carcinomas [4,7].

EUS-guided tissue biopsy remains the only method of distinguishing between a PM and a primary pancreatic cancer, with a sensitivity of 80–90% and a specificity of 100% and minimal morbidity, including hemorrhage and infection [7,12,18,19,20,44]. The cytological examination of PMs can mimic that of primary pancreatic carcinoma [18]. Imaging studies can only accurately assess the stage of the disease, while histopathological examination can distinguish between a primary tumor and pancreatic metastatic lesions [14].

Although squamous cell pancreatic carcinoma (SCC) is very rare, differentiation between a primary SCC of pancreas and a metastatic ESCC pancreatic tumor by microscopy can be challenging. The World Health Organization recommends categorizing a primary SCC as a component of adenosquamous cell carcinoma, rather than as a pure SCC [45,46,47,48]. If the patient has already been diagnosed with an SCC, the probability of a PM should be first considered and histopathological and immunohistochemical examination is needed [2,6,45,46].

There have been attempts to explain the possible mechanisms of esophageal cancer’s metastasis to the pancreas, but since most of the papers are case reports, common pathways are difficult to identify [1,4]. Metastasis of esophageal carcinoma to unexpected organs has been attributed to esophageal anatomical features such as the absence of serosa, the presence of periesophageal adventitia and multiple arterial resources [1].

The three proposed ways of spread are local lymphogenic, local venous and systemic hematogenic [1,4,42,43]. Through the veins, the tumor cells can pass into the vena cava, then into the lung, which explains the lung metastases, or into the portal system, which explains the liver metastases [1]. Unexpected metastasis can be explained by arterial dissemination, with metastasis to the pancreas being of hematogenous systemic origin [1,42,43]. If the hematogenous spread is predominant, metastases can be diffusely distributed in the pancreas, with no preference for an anatomical part of the organ [42,43]. Given the small amount of blood flowing through 120–180 g of pancreatic tissue, it is very unlikely that all embolized tumor cells will be transported exclusively to the pancreas [33].

Sellner et al. used the term “seed and soil mechanism” coined by Paget in 1882 [42,43]. According to this theory, after the systemic dissemination of tumor cells from a primary carcinoma, not all cells become metastatic [42,43]. The tumor cells can develop into clinically manifest metastasis only if the metastatic tumor cells (seeds) and host organ cells (soil) have distinct biological properties that exactly match each other. If they do not, metastatic cells will be destroyed by immune mechanisms [42,43]. The mechanism that causes solitary PMs is unknown [42]. Some studies describe a role for miRNAs in the genesis of PMs [49]. They can control the behavior of circulating cells by their ability to inhibit many target genes involved in various stages of the metastatic cascade (e.g., epithelial–mesenchymal transition, migration of tumor cells and metastasis settlement [42,43,49]. The variable interactions of specific miRNAs in different tumor cells determine multiple capacities for metastasis, increasing the chances that one of the embolized tumor cells will exactly match the properties of the targeted organ [42,43,49].

Organotrophy can be expected if the early metastatic phase involves the interaction of tumor cell and host organ properties [42,48,49]. Organotrophy can be explained by the interaction of a chemokine receptor on the surface of the tumor cell and a suitable ligand in the host organ [42,43,49]. This is a necessary condition for the activation of signal transduction pathways, which are critical in the migration, angiogenesis, invasion and proliferation of tumor cells [42,48]. The interaction takes place only in tissues where the receptors and ligands correspond exactly because the chemokine receptors are specific to the tumor cell, with the type and level of the ligand being specific to the host organ [42,43].

Solid tumors weaken the humoral and cellular defense mechanisms of potential host organs [42,43]. Thus, subsequent embolization and colonization of tumor cells can occur successfully, and metastasis can form in any organ [42].

Although, for many years, the resection of a metastatic tumor in the has been was avoided, due to it having high rates of morbidity and mortality, recent data have shown that surgical treatment of PMs is safe, feasible and can provide survival benefits for well-selected cases [2,21,22,23,24,25,26]. Patients who can benefit from surgery and have no metastasis in other organs should be considered good candidates for pancreatic resection [25,50,51,52,53].

Most patients with PMs of the ESCC are not candidates for surgical resection because they are usually found during diagnosis to have widespread systemic disease [5]. PM resection provides a good prognosis with a good long-term survival rate only for solitary metastases [2,5,6,7,8]. Patients with PM of ESCC may benefit from pancreatic metastasectomy if they meet the following criteria: the primary cancer has been treated with favorable results, without local recurrence; the PM is solitary and resectable; and the patient can tolerate pancreatectomy [2,8].

The effectiveness of metastasectomy depends on the biology of the primary cancer and whether the metastasis is synchronous or metachronous [8]. The use of pancreatectomy in the treatment of metastatic RCC is accepted, even in patients with multiple PMs, if the metastasectomy is radical [12,13,14,23,29,30,42,50,51,52,53,54,55].

Pancreatic resection, in patients with RCC and PMs, leads to better survival in comparison with other nonrenal cancers (ovarian, lung, sarcoma, melanoma, colorectal, breast and others) [9,14,16,21,22,23,25]. In these tumors, surgery is mainly palliative, being recommended in selected cases and especially in solitary lesions [9,14,21,25,26,29,30]. The impact of pancreatectomy on PM from nonrenal primary cancer is still unclear and requires further study in the future due to the small number of cases available [9,21]. If radical, pancreatectomies for PMs should be considered as part of multidisciplinary patient management because they prolong patient survival and improve quality of life [9,21]. Some authors have reported favorable results and prognosis with the resection of PMs by ESCC, though the reports are limited due to the low number of cases of PMs caused by esophageal carcinoma [2,3,4,8,10]. Studying the literature, out of the nine published cases of PMs caused by ESCC, in only five cases was pancreatectomy performed [2,3,8,9,10]. In three cases, pancreatectomy was carried out for solitary metachronous metastasis [2,8,9], and in two cases, it was performed for synchronous solitary metastasis [3,10]. In all five cases, distal pancreatectomy was performed.

With respect to the surgical technique used, standardized pancreatic resection is usually adapted to the location of the tumor. Cephalic (partial) pancreaticoduodenectomy, distal pancreatectomy and total pancreatectomy are radical procedures that include regional lymphadenectomy [14,22,28,31,48]. Standard radical resection is recommended in PMs caused by RCC, especially if they are multiple, to prevent postoperative recurrence [28,29,51,54].

Atypical resection, in the form of enucleation, central pancreatectomy or limited pancreatic resection, aims to preserve the parenchyma and usually does not include lymphadenectomy [27,51,54]. Atypical resection has a high risk of recurrence and postoperative complications (pancreatic fistula) compared to typical resection [14,21,22,53].

Intraoperative ultrasound is very useful in choosing the most appropriate surgical procedure, doing so by highlighting the presence of several pancreatic lesions and the distance between the metastasis and the Wirsung duct [19]. The surgical strategy needs to be adapted for each case to achieve an R0 resection, ensuring the absence of residual tumors in the pancreatic parenchyma [22]. Aggressive surgery for PMs should be considered if surgical resection is curative R0 [52,53,55]. This is not only to make a definitive diagnosis in order to select for further chemotherapy but also for improving survival, even in the presence of recurrences and metastasis [52,54,55]. The resection of metachronous PMs gives better results than the resection of synchronous PMs [50].

The role of lymphadenectomy in patients with PMs is controversial. It needs to be performed when PMs are accompanied by metastasis in regional lymph nodes [23,25]. Lymph node dissection may be omitted when the metastatic disease does not spread to the peripancreatic lymph nodes, which consequently reduces the extent of surgical excision, thus decreasing the complication rate [27,50,53]. The treatment of PMs is, however, multimodal. Surgery is only one option, and pancreatectomy can provide good palliative care in some patients [22,26].

For many years, surgical resection has been the only therapeutic option for PMs, but lately targeted therapies with multi-tyrosine kinase inhibitors, MTOR inhibitors, VEGF inhibitors and immunotherapies have been shown to be extremely effective [42,43].

The successful introduction of immunomodulatory therapy with immune checkpoint inhibitors such as anti-PD-1 (Nivolumab) or anti-CTLA-4 (Ipilimumab) has demonstrated the importance of immunostimulation in patients with metastatic RCC, but there are no detailed data about their specificity for PM [42,43]. Most studies have included metastatic RCCs, which are characterized by indolent biology, increased angiogenesis and an uninflamed stroma. These are likely to be the basis for good prognosis, sensitivity to antiangiogenic therapies and being refractory to immune checkpoint inhibitors such as Nivolumab [48]. These data suggest that metastatic organotropism may be an indicator of a particular biology with prognostic and treatment implications [48].

Recent studies have suggested that therapy with tyrosine kinase inhibitors for RCC with PMs had long-term efficacy comparable to surgery, but survival without residual disease was better with the surgical approach [21]. Another study showed that the survival of patients with metastatic RCC treated via non-operative management for 5 years was lower compared to that after surgical resection [54,55]. Pancreatectomy may provide a survival benefit in patients with PMs from RCC, even in the era of tyrosine kinase inhibitors for recurrent RCC. However, a combination of pancreatectomy with chemotherapy may be needed to improve the prognosis [21,23,30].

Based on what we found in the literature, the effectiveness of modern chemotherapy does not differ from that of surgical treatment [30]. Radical surgery, especially in cases with RCC PMs, gives patients a chance to heal or survive a long time without recurrence and avoid the long-term consequences of chemotherapy [30]. The effectiveness of tyrosine kinase inhibitors is proven and is recommended in patients who cannot have surgery for various reasons or are at increased risk of postoperative complications [23].

## 4. Conclusions

Pancreatic metastasis of esophageal squamous cell carcinoma is extremely rare. Pancreatic metastasis may develop several years after the treatment of the primary lesion. The diagnosis of metastasis is difficult, requiring histopathological and immunohistochemical examination. Pancreatic metastasectomy is safe and feasible in well-selected patients and is associated with acceptable long-term survival.

## Figures and Tables

**Table 1:** Reported cases of pancreatic metastasis from esophageal carcinoma.

Nr	Authors (Year of Publication)	Age (Years)	Sex	Therapy of ESCC	Interval between ESCC and Pancreatic Metastasis (Months)	Surgery of Pancreatic Metastasis	Follow-Up (Months)	Localization of Pancreatic Metastasis
1	Esfehani et al. (2011) [9]	59	F	S + AT	48	DP	4	body-tail
2	Park et al. (2013) [3]	58	M	S + AT	Synchronous	DP	6	tail
3	Sawada et al. (2013) [5]	73	M	No	Synchronous	No	3	body
4	Okamoto et al. (2014) [2]	68	M	NAT + S	24	DP	9	body
5	Koizumi et al. (2019) [8]	81	F	S	132	DP	24	tail
6	Yoon et al. (2019) [4]	67	M	AT	Synchronous	No		head
7	Ahmadi Somaghian et al. (2021) [6]	78	F	AT	Synchronous	No	9	body-head
8	Zang et al. (2021) [7]	67	M	AT	Synchronous	No	1	body-tail
9	Denda et al. (2023) [10]	54	M	S + AT	Synchronous	DP	9	tail
10	Present case (2024)	75	M	S	67	No	7	body-head

Abbreviations: F—female; M—male; S—surgery; DP—distal pancreatectomy; AT—adjuvant therapy; NAT—neoadjuvant therapy.

## Data Availability

The data presented in this study are available on request from the corresponding author.
